# Taxonomy of anxiety disorders—a comparison of ICD‑10 and ICD‑11

**DOI:** 10.1007/s00115-025-01842-6

**Published:** 2025-07-29

**Authors:** Katharina Domschke, Peter Zwanzger

**Affiliations:** 1 Department of Psychiatry and Psychotherapy, Medical Center—University of Freiburg, Faculty of Medicine, University of Freiburg, Hauptstraße 5, 79104 Freiburg, Germany; 2 kbo-Inn-Salzach-Hospital, Department of Psychosomatic Medicine, Competence Center for Anxiety, Wasserburg am Inn, Germany; 3 Department of Psychiatry and Psychotherapy, Ludwig Maximilians University Munich, Munich, Germany

**Keywords:** Panic disorder, Generalized anxiety disorder, Social anxiety disorder, Separation anxiety disorder, Selective mutism, Panikstörung, Generalisierte Angststörung, Soziale Phobie, Störung mit Trennungsangst, Selektiver Mutismus

## Abstract

With the introduction of the 11th revision of the World Health Organization International Statistical Classification of Diseases and Related Health Problems (ICD-11), structural and content-related adjustments to the diagnostic guidelines for anxiety disorders were made, which are presented in this review article. Previously classified as “phobic disorders” and “other anxiety disorders” within the group “neurotic, stress-related, and somatoform disorders”, in ICD-11 “anxiety- or fear-related disorders” now constitute a separate group. The core diagnoses of agoraphobia, social anxiety disorder, specific phobia, panic disorder and generalized anxiety disorder are retained, with the modification that agoraphobia and panic disorder can now be diagnosed separately and comorbidly. Within the framework of the lifespan perspective, separation anxiety disorder and selective mutism have been moved to the group “anxiety- or fear-related disorders”. The diagnosis “mixed anxiety and depressive disorder” is now classified as “mixed depressive and anxiety disorder” in the group “affective disorders”. In accordance with the 5th edition of the Diagnostic and Statistical Manual of Mental Disorders (DSM‑5), it is possible to code isolated panic attacks in addition to other mental or somatic disorders. Overall, ICD-11 follows the DSM‑5 classification of anxiety- and fear-related disorders in many respects. Furthermore, the omission of subcategorizations and a precise minimum number of required symptoms simplify the diagnostic criteria. Future studies will need to address questions regarding the diagnostic accuracy, clinical practicability and further operationalization of the ICD-11 diagnostic criteria for anxiety- or fear-related disorders.

## Abstract (de)

Mit Einführung der 11. Revision der Internationalen statistischen Klassifikation der Krankheiten und verwandter Gesundheitsprobleme der World Health Organization (ICD-11) wurden strukturelle und inhaltliche Anpassungen der diagnostischen Richtlinien für Angststörungen vorgenommen, die in diesem Übersichtsartikel dargestellt werden. Die bisher in der Gruppe „Neurotische, Belastungs- und somatoforme Störungen“ verorteten „Phobischen Störungen“ bzw. „Anderen Angststörungen“ werden in der ICD-11 als „Angst- oder furchtbezogene Störungen“ in einer eigenen Gruppe zusammengefasst. Dabei wird an den zentralen Diagnosen Agoraphobie, soziale Angststörung, spezifische Phobie, Panikstörung und generalisierte Angststörung festgehalten, wobei Agoraphobie und Panikstörung nun getrennt voneinander und komorbid vergeben werden können. Im Rahmen der Lebensspannenperspektive wurden zudem die Störung mit Trennungsangst und der selektive Mutismus in die Gruppe der „Angst- oder furchtbezogenen Störungen“ aufgenommen. Die Diagnose „Angst und depressive Störung, gemischt“ wird nun als „Gemischte depressive Störung und Angststörung“ der Gruppe der „Affektiven Störungen“ zugeordnet. Analog der 5. Ausgabe des Diagnostic and Statistical Manual of Mental Disorders (DSM‑5) besteht die Möglichkeit, isolierte Panikattacken zusätzlich zu anderen psychischen oder somatischen Erkrankungen zu kodieren. Insgesamt folgt damit die ICD-11 im Bereich der Angst- oder furchtbezogenen Störungen in vielen Punkten der Klassifikation des DSM‑5. Darüber hinaus stellt der Verzicht auf Subkategorisierungen und eine exakte Mindestanzahl von Symptomen eine Vereinfachung der Diagnostik dar. Zukünftige Studien werden sich Fragen zur diagnostischen Genauigkeit, klinischen Praktikabilität und weiteren Operationalisierung der ICD-11 Diagnosekriterien für Angst- oder furchtbezogene Störungen widmen müssen.

In the 10th edition of the *International Statistical Classification of Diseases and Related Health Problems* (ICD-10), anxiety disorders (F40–F41) are listed in the group “Neurotic, stress-related and somatoform disorders”, which also includes “Obsessive-compulsive disorder”, “Reaction to severe stress, and adjustment disorders”, “Dissociative [conversion] disorders”, “Somatoform disorders” and “Other neurotic disorders”. In ICD-11, similar to the fifth edition of the *Diagnostic and Statistical Manual of Mental Disorders* (DSM-5), anxiety disorders are now contained in their own group and thus differentiated from “Obsessive-compulsive disorders”, “Disorders specifically associated with stress”, “Dissociative disorders”, and “Disorders of bodily distress or bodily experience”, which are listed in separate groups. The concept of neurosis has been abandoned.

The ICD-10 distinguishes between two categories of anxiety disorders: “Phobic disorders” (F40.-) and “Other anxiety disorders” (F41.-). In ICD-11, this subdivision has been omitted; instead, the group is referred to as “Anxiety- or fear-related disorders” (see Table 1; [15, 16]). Thus, while abandoning a structural differentiation, the linguistic distinction between anxiety disorders and “phobic” (now “fear-related”) disorders remains, reflecting the conceptual separation of anxiety and fear as proposed by, for instance, Freud, Heidegger and Kirkegaard, and as more recently substantiated at the neurobiological level [3].

In the transition from ICD-10 to ICD-11, the following structural modifications have been made (also see [2, 5, 6, 13]):

- With the introduction of the lifespan approach in ICD-11 and in the spirit of parallelization with DSM‑5, two disorders from the ICD-10 group “Behavioural and emotional disorders with onset usually occurring in childhood and adolescence” (F90-F98) were moved to the group of “Anxiety or fear-related disorders”: “Separation anxiety disorder of childhood” (F93.0) is no longer listed in the category “Emotional disorders with onset specific to childhood”, but is now classified as “Separation anxiety disorder” (6B05) parallel to other anxiety or fear-related disorders. Similarly, “Elective mutism” (F94.0), formerly included in the category “Disorders of social functioning with onset specific to childhood and adolescence”, is now referred to as “Selective mutism” (6B06) and classified as an “Anxiety or fear-related disorder”. Accordingly, “Phobic anxiety disorder of childhood” (F93.1) and “Social anxiety disorder of childhood” (F93.2) are now merged into the ICD-11 categories “Specific phobia” (6B03) and “Social anxiety disorder” (6B04), respectively [14].
- The classification of panic disorder and agoraphobia has been simplified in ICD-11 compared to ICD-10, in that both diagnoses can now be assigned separately and comorbidly.
- In analogy to a DSM‑5 “specifier”, isolated panic attacks that do not meet the diagnostic criteria for panic disorder can be coded in addition to anxiety disorders or other mental disorders in Chapter 21 “Symptoms, signs or clinical findings, not elsewhere classified” (MB23.H). Furthermore, the symptoms “anxiety” or “fear” can be coded as MB24.3 or MB24.A, respectively; the symptom “worry”, as MB24.H.
- In ICD-10, “Mixed anxiety and depressive disorder” (F41.2) is coded as “Other anxiety disorder”; in ICD-11, the entity “Mixed depressive and anxiety disorder” (6A73) has been assigned to the group “Mood disorders”, under the category “Depressive disorders.”
- In ICD-10, substance-induced anxiety disorders are listed in the group “Mental and behavioural disorders due to psychoactive substance use” (category: “Other mental and behavioural disorders”). In ICD-11, they are contained in the group “Disorders due to substance use or addictive behaviours” as “Certain specified mental or behavioural disorders”, e.g. “Alcohol-induced anxiety disorder” (ICD-10: F10.8/ICD-11: 6C40.71), “Cannabis-induced anxiety disorder” (ICD-10: F12.8/ICD-11: 6C41.71).
- “Organic anxiety disorder” (F06.4) is classified in the ICD-10 group “Organic, including symptomatic, mental disorders” (category: “Other mental disorders due to brain damage and dysfunction and to physical disease”). In ICD-11, “Secondary anxiety syndrome” (6E63) is contained in the group “Secondary mental or behavioural syndromes associated with disorders or diseases classified elsewhere”.

**Table 1:** Taxonomy of anxiety disorders: comparison between ICD-10 and ICD-11

ICD-10	ICD-11
Neurotic, stress-related and somatoform disorders	Anxiety or fear-related disorders
*F40: Phobic anxiety disorders*	
Agoraphobia (F40.0) – Agoraphobia without history of panic disorder (F40.00) – Panic disorder with agoraphobia (F40.01)	Agoraphobia (6B02)
Social phobias (F40.1)	Social anxiety disorder (6B04)
Specific (isolated) phobias (F40.2)	Specific phobia (6B03)
Other phobic anxiety disorders (F40.8)	–
Phobic anxiety disorder, unspecified (F40.9)	–
*F41: Other anxiety disorders*	
Panic disorder [episodic paroxysmal anxiety] (F41.0) – moderate (F41.00) – severe (F41.01)	Panic disorder (6B01)
Generalized anxiety disorder (F41.1)	Generalized anxiety disorder (6B00)
Mixed anxiety and depressive disorder (F41.2)	–
Other mixed anxiety disorders (F41.3)	–
–	Separation anxiety disorder (6B05)
–	Selective mutism (6B06)
Other specified anxiety disorders (F41.8)	Other specified anxiety or fear-related disorders (6B0Y)
Anxiety disorder, unspecified (F41.9)	Anxiety or fear-related disorders, unspecified (6B0Z)

In terms of content, the diagnostic criteria of anxiety disorders were modified linguistically and conceptually, as detailed here [15, 16]. In general, in addition to the active avoidance of feared situations or objects, the ICD-11 explicitly states that these can also be endured with intense fear or anxiety. This aspect is contained in the ICD-10 preamble to phobic disorders and, in more recent editions, in the diagnostic criteria of the individual disorders as well. Furthermore, the ICD-11 systematically addresses the lifespan perspective as well as gender-related and culture-specific aspects.

## Generalized anxiety disorder (6B00)

The ICD-11 specifies excessive concern regarding particular areas of life, i.e. family, health, finances, and school or work. A precise number of symptoms as defined in ICD-10 (“at least four of the 14 symptoms of a panic attack, including at least one autonomic symptom”) is no longer required. The requirement in ICD-10 to exclude a diagnosis if the criteria for specific phobia, panic disorder, obsessive-compulsive disorder, or hypochondriacal disorder are met has been removed in ICD-11. The time criterion is no longer strictly fixed at “at least six months”.

## Panic disorder (6B01)

In ICD-11, the addition “episodic paroxysmal anxiety” has been removed in the interest of standardization with DSM‑5. Coding the degree of severity (see Table 1) is no longer possible. In contrast to ICD-10 (“at least four of 14 symptoms, including at least one autonomic symptom”), the ICD-11 does not require a minimum number of symptoms. In ICD-11, it is now explicitly stated that panic attacks are followed by persistent worry or concern about their recurrence or their perceived negative significance, or by behaviours that could prevent their recurrence. A time criterion is not specified. Furthermore, the ICD-11, in analogy to the DSM‑5, mentions nocturnal panic attacks and culture-specific forms such as “Ataque de nervios” or “Khyâl cap”.

## Agoraphobia (6B02)

In ICD-10, panic disorder and agoraphobia can be coded separately, and agoraphobia can also be coded with or without panic disorder (see Table 1). In ICD-11, agoraphobia and panic disorder are now clearly differentiated from one another and considered two fundamentally separate entities that can be diagnosed comorbidly. In contrast to ICD-10 (“at least two of the 14 symptoms of a panic attack, including at least one autonomic symptom”), a minimum number of symptoms is no longer required. Furthermore, in ICD-11 marked and persistent fear or avoidance no longer has to explicitly occur in at least two of four situations (“crowds; public places; traveling alone; traveling far from home”). In terms of content, the ICD-11 emphasizes that the central cognitive fear primarily concerns situations in which escape might be difficult or no help is available, and that negative consequences such as panic attacks, panic symptoms, or other disabling or embarrassing physical symptoms are feared. The time criterion in ICD-11 is set at “at least several months”.

## Specific phobia (6B03)

In ICD-11, fears do not have to be excessive, but they do have to be disproportionate to the actual risk posed by the specific object or situation, taking into account both accepted cultural norms and the specific environmental conditions to which the affected person is normally exposed. The differentiation of subtypes in ICD-10 (e.g. “animal type”, “blood, injection, injury type”, “situational type”) is no longer applicable in ICD-11. The time criterion in ICD-11 is set at “at least several months”.

## Social anxiety disorder (6B04)

In analogy to DSM‑5, “Social phobia” was renamed “Social anxiety disorder” in ICD-11. The core criteria remain largely unchanged, but now emphasize the cognitive fear of behaving in a way or displaying anxiety symptoms that could be negatively evaluated by others. The minimum number of symptoms required by the ICD-10 (“at least two of the 14 symptoms of a panic attack plus at least one of the following symptoms: blushing or trembling; fear of vomiting; urge or fear of urinating or defecating”) has been abolished in the ICD-11. In line with DSM‑5, culture-specific social anxiety disorders such as “Taijin kyofusho” are now taken into account. The time criterion in the ICD-11 is set at “at least several months”.

## Separation anxiety disorder (6B05)

In ICD-11, the criterion of childhood onset has been removed. Separation anxiety disorder is characterized by pronounced and developmentally inappropriate fear or concern regarding separation from specific attachment figures such as parents, caregivers or family members in childhood, or romantic partners or children in adulthood. The symptoms are not transient, i.e. they persist for at least several months (also see [1, 10]). The presence of affective disorders, selective mutism or social anxiety disorder is an exclusion criterion for the diagnosis of separation anxiety disorder in ICD-11.

## Selective mutism (6B06)

The criterion of childhood onset has been removed in the ICD-11. Furthermore, in line with DSM‑5, the disorder was renamed from “Elective mutism” to “Selective mutism”. Selective mutism is characterized by consistent selectivity in speaking, meaning that a child or adult has adequate language competence in specific social situations, typically at home, but consistently fails to speak in other situations, typically at school. The duration of the disorder is at least 1 month, but not limited to the first month of school (also see [9]). The presence of schizophrenia or autism spectrum disorder is an exclusion criterion for the diagnosis of selective mutism in ICD-11.

## Conclusion and outlook

- The conceptualization of anxiety and fear-related disorders in ICD-11 differs significantly in some respects from ICD-10 and is closely aligned with DSM‑5.
- The diagnoses “Panic disorder” and “Agoraphobia” can now be given separately and comorbidly.
- The diagnosis “Mixed anxiety and depressive disorder” is now coded as “Mixed depressive and anxiety disorder” and assigned to the group “Mood disorders”.
- The lifespan perspective is particularly noteworthy, with the inclusion of separation anxiety disorder and selective mutism into the group of anxiety disorders, thus taking into account the close epidemiological and aetiological relationship of these two entities with other anxiety- or fear-related disorders, as well as their relevance in adulthood.
- Accordingly, separation anxiety disorder will now have to be considered in adult patients with corresponding symptoms. For this purpose, the Adult Separation Anxiety Questionnaire (ASA-27) is available, also in German [11]. Differential diagnoses include generalized anxiety disorder and—according to ICD-10 nomenclature—dependent (F60.7) or anxious-avoidant (F60.6) personality disorder.
- Also, selective mutism should receive increased diagnostic attention in adulthood and should be differentiated from dissociative disorder in the form of psychogenic aphonia (ICD-10: F44.4; ICD-11: 6B60.5) or depressive stupor. On the somatic side, deafness, various neurological or otorhinolaryngological diseases leading to motor aphasia or aphonia, akinetic mutism, which is characterized by a severe disturbance of drive and motor function in general, or postoperative cerebellar mutism must be excluded [4].
- Initial studies indicate a higher diagnostic accuracy of ICD-11 criteria for anxiety disorders compared to ICD-10, particularly for generalized anxiety disorder [7].
- Future efforts will need to focus on further operationalizing ICD-11 diagnostic criteria for anxiety disorders in everyday clinical practice, such as the WHO Flexible Interview for ICD-11 (FLII-11; [8]) or the International Anxiety Questionnaire (IAQ; [12]).

## Declarations

For this article no studies with human participants or animals were performed by any of the authors. All studies mentioned were in accordance with the ethical standards indicated in each case.

The supplement containing this article is not sponsored by industry.
